# Prioritizing cancer-related genes with aberrant methylation based on a weighted protein-protein interaction network

**DOI:** 10.1186/1752-0509-5-158

**Published:** 2011-10-11

**Authors:** Hui Liu, Jianzhong Su, Junhua Li, Hongbo Liu, Jie Lv, Boyan Li, Hong Qiao, Yan Zhang

**Affiliations:** 1 College of Bioinformatics Science and Technology, Harbin Medical University, Harbin, China; 2 The Second Affiliated Hospital, Harbin Medical University, Harbin, China; 3 The Academy of Fundamental and Interdisciplinary Science, Harbin Institute of Technology, Harbin, China

## Abstract

**Background:**

As an important epigenetic modification, DNA methylation plays a crucial role in the development of mammals and in the occurrence of complex diseases. Genes that interact directly or indirectly may have the same or similar functions in the biological processes in which they are involved and together contribute to the related disease phenotypes. The complicated relations between genes can be clearly represented using network theory. A protein-protein interaction (PPI) network offers a platform from which to systematically identify disease-related genes from the relations between genes with similar functions.

**Results:**

We constructed a weighted human PPI network (WHPN) using DNA methylation correlations based on human protein-protein interactions. WHPN represents the relationships of DNA methylation levels in gene pairs for four cancer types. A cancer-associated subnetwork (CASN) was obtained from WHPN by selecting genes associated with seed genes which were known to be methylated in the four cancers. We found that CASN had a more densely connected network community than WHPN, indicating that the genes in CASN were much closer to seed genes. We prioritized 154 potential cancer-related genes with aberrant methylation in CASN by neighborhood-weighting decision rule. A function enrichment analysis for GO and KEGG indicated that the optimized genes were mainly involved in the biological processes of regulating cell apoptosis and programmed cell death. An analysis of expression profiling data revealed that many of the optimized genes were expressed differentially in the four cancers. By examining the PubMed co-citations, we found 43 optimized genes were related with cancers and aberrant methylation, and 10 genes were validated to be methylated aberrantly in cancers. Of 154 optimized genes, 27 were as diagnostic markers and 20 as prognostic markers previously identified in literature for cancers and other complex diseases by searching PubMed manually. We found that 31 of the optimized genes were targeted as drug response markers in DrugBank.

**Conclusions:**

Here we have shown that network theory combined with epigenetic characteristics provides a favorable platform from which to identify cancer-related genes. We prioritized 154 potential cancer-related genes with aberrant methylation that might contribute to the further understanding of cancers.

## Background

Cancer is a complex multi-gene disease. For a long time, gene mutation has been considered to be related to cancer. A number of oncogenes and tumor suppressor genes linked to mutations have been shown to drive the neoplastic process by increasing tumor cell numbers [1-3]. However, with progress in the understanding of cancer and the ongoing development of epigenetics, it has been reported that aberrant DNA methylation events are involved in many types of cancers [4]. Genome-wide hypomethylation and region-specific CpG island promoter hypermethylation are a hallmark of the cancer epigenome [5]. Thus the mechanisms that drive cancer development and progression cannot be effectively uncovered by studying only genetic factors. For a more profound understanding epigenetic characteristics must also be examined.

Abnormal DNA methylation has been found in many different tumors, including brain tumor, breast cancer, and prostate cancer. DNA hypomethylation is often considered to activate oncogenes. DNA hypermethylation of the promoter region, on the other hand, may initiate the silencing of tumor suppressor genes. It has been reported that the overexpression of *FEN1 *(flap endonuclease) in breast and other cancers is associated with CpG island hypomethylation of the promoter region in the tumor cells [6]. Similarly, in a recent study by Sun et al., it was found that the promoter of *TKTL1 *(transketolase-like 1) had a high frequency of hypomethylation which induced the overexpression of the gene in head and neck squamous cell carcinoma (HNSCC) [7]. *BRCA1 *is a well-characterized tumor suppressor gene which codes for proteins that have important roles in the regulation of the cell cycle and in the apoptosis of tumor cells. In breast and ovarian cancers, *BRCA1 *is inactivated by hypermethylation within the promoter region of the gene [8].

Thus, as the number of epigenetic studies gradually increase, the importance of DNA methylation in cancer research is being recognized. Simultaneously, a series of methods for DNA methylation detection have been developed. The earliest DNA methylation detection methods were mainly single-gene sequence-specific methods such as methylation sensitive restriction endonucleases [9], methylation specific PCR [10], and combined bisulfite restriction analysis [11]. Subsequently, high-throughput genomic DNA methylation detection methods were developed like, for example, the large-scale microarray and sequencing technologies [12,13]. Numerous studies of gene methylation have used traditional experimental methods to generate large amounts of methylation data; more recently, a large number of genome-wide DNA methylomes have been generated through the traditional methods being combined with the high throughput technologies.

Some cancers have been identified as different subtypes based on the methylation level of CpG islands [14] and cancer-related genes have been identified by their epigenetic variations. Loss et al. prioritized genes with epigenetic regulation in 45 breast cancer cell lines by their ranked methylation-expression association using logistic regression, and identified 58 genes as epigenetically regulated genes in the breast cancer cell lines [15]. Some cancer-related genes have been identified by combining epigenetic characteristics and network theory. Network theory provides a platform for the systematic study of diseases [16-19]. The prioritization of cancer-related genes has also been widely studied using network theory. Most network theoretical approaches are based on the assumption that cancer-related genes participate in common functional modules including protein complexes, molecular pathways and developmental processes, and may have the same or similar functions that are involved in the development of cancers. Charles et al. established a weighted function network composed of human genes, and ranked the related genes to 110 different diseases (including cancer). This study revealed the recondite relationship between diseases with quite different phenotypes [20]. In a study by Cui et al., a manually curated human signaling network was constructed and a set of cancer mutated genes and a set of cancer-associated methylated genes were mapped into the signaling network. These researchers found that methylated genes were mainly enriched in negative regulatory loops encoding tumor suppressors in cancer cells [21]. Thus, they successfully developed an approach to identify cancer-related genes that could be used as biomarkers of cancers from high-throughput data.

A human protein-protein interaction network, constructed using a machine learning method, has been shown to be of benefit when applied to the study the disease-related genes using network theory [22]. Here, we report the construction of an integrated and weighted network using protein-protein interaction (PPI) data and its correlation with DNA methylation to provide a comprehensive approach for prioritizing the cancer-related genes with abnormal methylation from genome and epigenome data.

## Results

**Figure 1:**
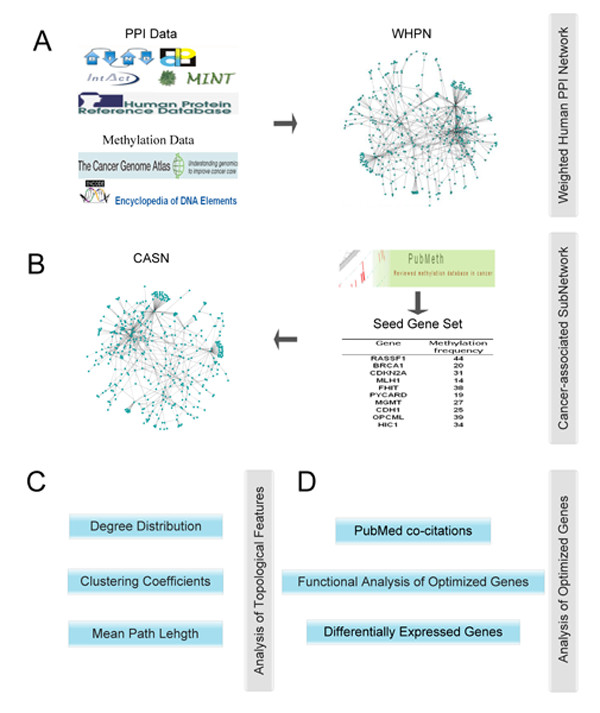
**Workflow used in the present study**. (A) The PPI and DNA methylation data sources used in the construction of WHPN. (B) The acquirement of seed genes and the construction of CASN. (C) Analysis of the topological features of WHPN and CASN. (D) Analysis of the optimized genes using the GO and KEGG enrichment analysis, SAM and PubMed co-citations.

In this study, the workflow is shown in Figure 1. It consists of four major stages: (A) the construction of WHPN, a weighted human PPI network by integrating DNA methylation and protein-protein interaction features, (B) the formation of CASN, a cancer-associated subnetwork extracted by seed genes which from PubMeth, (C) the analysis of topological features between the two networks, (D) the prioritization cancer-related genes with aberrant methylation as optimized genes and the analysis of the optimized genes.

### The weighted human PPI network and cancer-associated subnetwork

**Figure 2:**
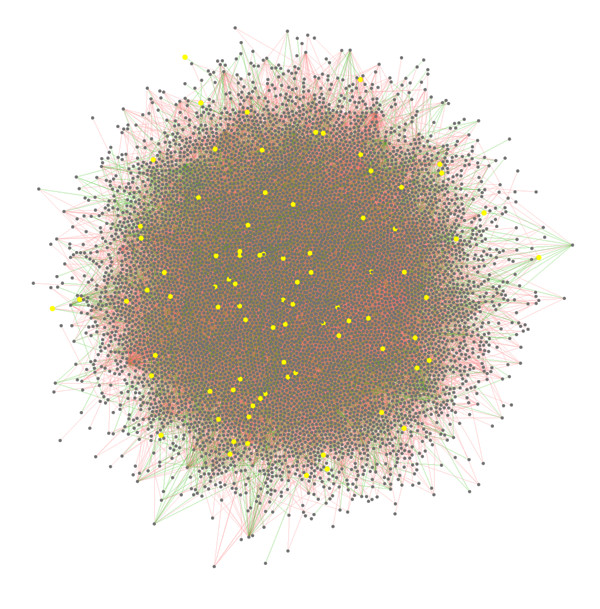
**Weighted human protein-protein interaction network (WHPN)**. There are 7840 nodes and 17671 linkages in WHPN. The nodes represent genes and the linkages represent protein-protein interactions. The yellow nodes represent the seed genes; the red linkages represent positive correlations and the green linkages represent negative correlations; the line width represents the weight of methylation correlation.

Protein-protein interaction (PPI) networks can be more perspicuous for the representation of the complex relationships between large numbers of elements and better at depicting the structure and function of the elements. In this study, we used a PPI network to understand the DNA methylation patterns present in the development and progression of cancers. We constructed the network by integrating DNA methylation and protein-protein interaction features to prioritize cancer-associated genes. The genes were used as nodes and the correlations of DNA methylation among genes were used as the linkage weight. The linkages whose methylation correlations were below the threshold are removed. The weighted human PPI network (WHPN) that we built contained 17617 interaction pairs covering 7840 human genes (see Methods for details) (Figure 2).

**Figure 3:**
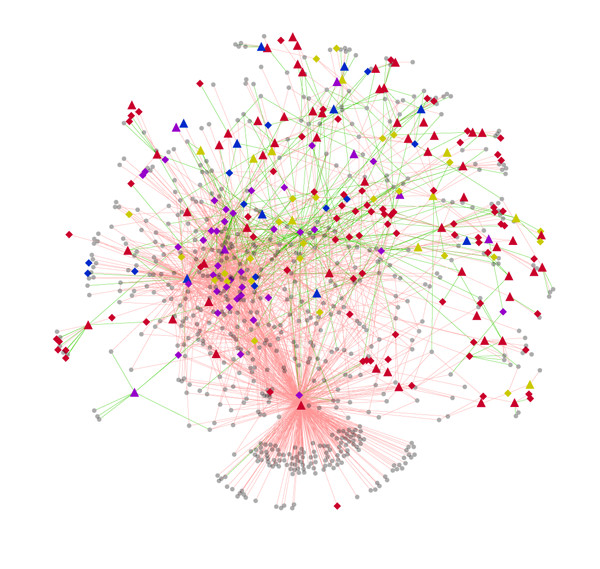
**Cancer-associated subnetwork (CASN)**. There are 857 nodes and 2333 linkages in CASN. The red linkages represent positive correlations and the green linkages represent negative correlations; the line width represents the weight of methylation correlation. The triangle nodes represent the seed genes and the diamond nodes represent the optimized genes; there are 84 seed genes and 154 optimized genes. The red nodes represent genes related to one type of cancer, the blue nodes represent genes related to two types of cancers, the yellow nodes represent genes related to three types of cancers and the purple nodes represent genes related to all the four types of cancers.

We identified 127 seed genes for the four types of cancers (glioblastoma, ovarian cancer, hepatocellular carcinoma and leukaemia) from PubMeth using text mining (see Method for details). The set of seed genes were classified into four types according to how many types of the four cancers the seed genes are associated with (Additional file 1). The seed genes were mapped to WHPN and a subset of 84 seed genes was obtained. In the set of 84 seed genes, 16 genes were for glioblastoma, 30 were for ovarian cancer, 41 were for hepatocellular carcinoma, and 48 were for leukemia. Using the seed genes, the cancer-associated subnetwork (CASN) was extracted from WHPN. CASN contains the seed genes and the genes which connect with the seed genes in WHPN comprising 857 genes (nodes) and 2333 interaction pairs (linkages) (Figure 3).

### Comparison of the topological features of WHPN and CASN

The topological features for WHPN and CASN were computed. CASN should have a network structure that is different from WHPN, which should show the specific genetic and epigenetic relations between genes in cancers.

**Figure 4:**
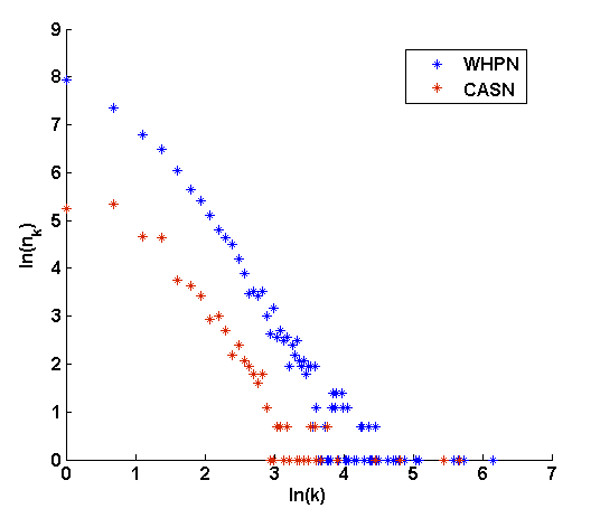
**The degree distributions of WHPN and CASN**. The degree distributions of WHPN (blue star) and CASN (red star) are shown. K is the degree of the nodes and the x axis is the ln transformed degree ln(k). The y axis is the ln transformed number of nodes for which the degree is k, ln(n_k_).

The average degrees for WHPN and CASN were 4.51 and 5.44, respectively, showing that CASN was much closer than WHPN. CASN was also more highly connected when compared with the 1000 randomly simulated subnetworks sampled the same number of nodes as in CASN from WHPN (Wilcoxon rank sum test, P < 1.0912E-200, FDR = 0.01). When the degree distributions of WHPN and CASN were compared (Figure 4), both WHPN and CASN followed a power-law distribution. The r values for the two networks were also obtained; r_WHPN _= 2.2178 for WHPN and r_CASN _= 1.8862 for CASN. A network which has a power-law degree distribution is generally called a scale-free network [23]. In both networks, there were a few nodes with high connectivity, referred to as Hub nodes; however, most nodes had low connectivity.

**Figure 5:**
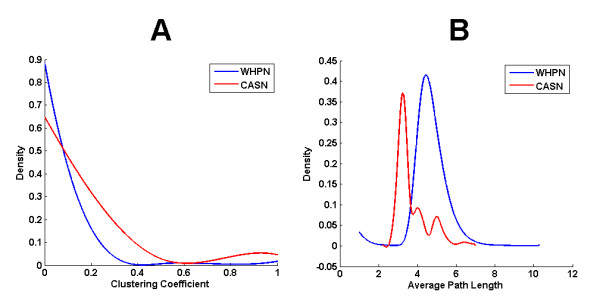
**Probability density distributions of the clustering coefficient and average path length for WHPN and CASN**. The blue and the red lines are for WHPN and CASN, respectively. (A) The decline for CASN is gentler than for WHPN. The x axis is the clustering coefficient with a range of 0 to 1. The probability density of the clustering coefficient is on the y axis. (B) Average path length is shown on the x axis and the probability density of average path length is on the y axis.

By analyzing the clustering coefficient of each node in WHPN and CASN, we found that CASN had a higher clustering coefficient than WHPN (Figure 5A). Most of the nodes in WHPN had lower clustering coefficients (clustering coefficient for WHPN, 0.0503) than the nodes in CASN (clustering coefficient for CASN, 0.1410). The clustering coefficient of CASN was also compared with the 1000 randomly simulated subnetworks sampled the same number of nodes as in CASN from WHPN (Wilcoxon rank sum test, P < 3.4408E-078, FDR = 0.01).

The average path length and the probability density distribution of the average path length were also compared for the two networks. The results showed that CASN had a shorter average path length (average path length for CASN, 3.6902) than WHPN (average path length for WHPN, 4.7335) (Figure 5B). As for the other topological features, the average path length of CASN was significant when compared with the average path lengths of the 1000 randomly simulated subnetworks (Wilcoxon rank sum test, P < 1.7703E-006, FRD = 0.01). The network diameters for WHPN and CASN were 15 and 10, respectively.

Although the topological features of CASN were significantly different from those of WHPN and the random subnetworks, to confirm the significance of our results, we used the topology-matched random subnetworks that were generated as described in the Method section. Compared with the topology-matched random subnetworks, the topology features of CASN were all statistically significant (Wilcoxon rank sum test, P_degree _< 1.9049E-095, P_clustering coefficient _< 1.7775E-042, P_average path length _< 2.0008E-091, FDR = 0.01). We also generated 1000 random networks by keeping the same number of nodes and connections as in CASN. We found that, compared with the three different random subnetworks, the degree and clustering coefficient for CASN were much significant those of the three random subnetworks, suggesting that the nodes in CASN were not randomly distributed (Additional file 2).

The calculated topological features of the networks indicate that CASN had a more densely connected network community than WHPN. It is generally believed that the mutant proteins contribute to diseases with similar phenotypes directly or indirectly interact and cancer is considered to be the result of the deregulation of some interrelated pathways. So, if a gene in the network is close to a cancer gene, then that gene is likely to be involved in some of the events that lead to the cancer. Further, the genes in CASN were also related to cancer through their abnormal methylation. Thus, the CASN genes may be involved in the same or similar biological processes as the seed genes, which may in turn be display changes of methylation levels in cancers.

### Prioritizing the cancer-related genes with aberrant methylation

To further investigate the proposed hypothesis that the genes that were close in the CASN network may have similar methylation levels, we selected 773 genes that were connected with the seed genes in CASN as candidate genes. The 773 candidate genes were assessed to prioritize the cancer-related genes with aberrant methylation that had not been detected in CASN before. Using the neighborhood-weighting decision rule [20], every candidate gene was scored to measure the possibility that the methylation state of the candidate genes varied in cancers. The candidate genes whose scores were larger than any of the 1000 simulated scores were identified as the optimized genes (See Methods for details). Finally, 154 CASN genes were prioritized using the seed genes and neighborhood-weighting decision rule (Additional file 3). The optimized genes are also classified as four types according to the number of cancer types that the genes connected; 79 optimized genes for type I, 12 for type II 26 for type III and 37 for type IV (Additional file 4).

### Analysis of Hub nodes and optimized genes

**Figure 6:**
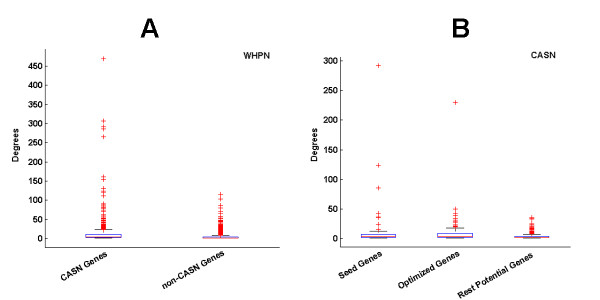
**Distributions of degree for the genes in WHNP and CASN**. (A) The CASN and non-CASN genes in WHPN are on the x axis; (B) The seed genes, optimized genes and rest potential genes in CASN are on the x axis. The y axis represents the degrees for these nodes. For the box plot, the top bar (black) is the lowest point within the 1.5 interquartile from the lower quartile, and the bottom bar (black) is the highest point within the 1.5 interquartile of the upper quartile, the top of box (blue) is the upper or third quartile, the bottom of box (blue) is the lower or first quartile, the middle bar is the median value. Pluses (red) are possible outliers.

The genes in WHPN were divided into two types, CASN gene set and non-CASN gene set. Overall, the connectivity of the CASN genes was higher than that of the non-CASN genes (Wilcoxon rank sum test, P = 2.6270E-145, Figure 6A, Additional file 5). The degrees for the CASN genes were in the range 1 to 470, and for the non-CASN genes the range was only from 1 to 116. The percentile 50 of degrees in CASN genes and non-CASN genes were 5 and 2, respectively (Figure 6A, Additional file 6). We found that, in WHPN, the top 10 genes with the largest degrees were all CASN genes (Additional file 5).

CASN genes were divided into three types: seed gene set, optimized gene set and rest potential gene set. The percentile 50 of degrees in the seed gene set, the optimized gene set and the rest potential gene set were 3.5, 4 and 2, respectively (Figure 6B, Additional file 7). The results showed that the seed genes and the optimized genes had higher degrees than the rest potential genes (Wilcoxon rank sum test, P_seed genes and rest potential genes _= 2.3400E-002, P_optimized genes and rest potential genes _= 5.4202E-004 and P_seed genes and optimized genes _> 0.1). Of the top 10 genes with the largest degrees in CASN, 5 were seed genes, 4 were optimized genes and only one was a rest potential gene which was ranked 10 (Additional file 5). Four of the optimized genes (*MAX*, *E2F4*, *EP300*, *MAPK8*) were all in the top 10 genes with the highest degrees in WHPN.

The gene, MYC associated factor X (*MAX*), with the second highest number of degrees in CASN and the highest number of degrees (470) in WHPN, was prioritized as an optimized gene. The MAX protein coded by this gene is a member of the basic helix-loop-helix leucine zipper (bHLHZ) family of transcription factors. MAX usually forms heterodimers with other family members such as Mad, Mxi1 and Myc. Myc is an oncoprotein involved in cell proliferation, differentiation and apoptosis [24]. Therefore, MAX may also participate in the same or similar biological processes that affect the development and progression of cancers. In CASN, some Hub nodes were prioritized as cancer-related genes with aberrant methylation. Other studies have shown that cancer-related proteins often have high connectivity that are usually considered to be the Hub notes in networks, rather than the peripheral notes [25,26]. The optimized genes in our study tended to have high connectivity, suggesting critical roles for them in important biological processes and in the deregulation of genes during the development and progression of the cancers.

### Analysis of GO categories and KEGG pathways

The GO function enrichment analysis of the CASN genes indicated that these genes were mainly enriched in the GO terms of regulation of programmed cell death and apoptosis, terms which are relevant of tumors (Additional file 8). The non-CASN genes, on the other hand, were mainly involved in the biological processes of regulation of protein RNA metabolic processes and cell proliferation (Additional file 8). Of the many significant GO terms listed for the non-CASN genes, only some of them were related to programmed cell death and apoptosis and they were significantly lower for the non-CASN genes than for the CASN gene set. These findings revealed that the CASN genes were much more closely related to cancers than the non-CASN genes. In the subsequent analysis, we focus on the CASN gene set.

The seed genes are mainly involved in GO biological processes associated with the regulation of the apoptosis and programmed cell death (Additional file 9). The seed genes were selected because they may be methylated aberrantly; thus, the abnormal methylation may induce their involvement in apoptosis and programmed cell death, affecting the development and progression of cancers [27,28]. We also found that the optimized genes were mainly enriched in the GO terms involving regulation of gene expression, regulation of transcription and in the biological processes of cell apoptosis and programmed cell death (Additional file 9). Apoptosis is a basic biological phenomenon that may have a direct or indirect relationship with many diseases, such as cancers and autoimmune diseases [29]. Apoptosis is negatively regulated in cancers and the disruption of apoptosis is involved in the initiation of cancers. These biological processes might be deregulated by the aberrant methylation of the optimized genes, thus affecting the process of cancers.

The CASN and non-CASN gene sets in WHPN were next analyzed for KEGG pathway enrichment. The findings revealed that the CASN genes were enriched in many cancer and cancer-associated pathways (Additional file 10); the non-CASN genes were not enriched in any cancer pathway. We investigated the three gene groups in CASN in the same way as we did for the GO enrichment analysis. The results for the optimized genes and seed genes are shown in Additional file 11. The seed genes were annotated to 12 of the 15 cancer pathways in the KEGG database. Two of the other pathways, the P53 signaling pathway and the Wnt signaling pathway, are closely related to cancers [30-32]. The optimized genes were also annotated to cancer pathways, and to the Notch signaling pathway and the cell cycle in KEGG. Some studies have shown that abnormalities of the Notch signaling pathway are linked to breast carcinogenesis, T cell malignancies, neuroblastoma, myeloid leukemia and lung cancer [33,34]. Our results also show that the optimized genes may be linked to cancers, suggesting that the abnormal methylation levels affect the development and progression of cancers.

Finally, the GO and KEGG enrichment analysis for the rest potential genes indicated that these genes may also be enriched in some cancer-related GO biological processes and in some KEGG cancer- related pathways (Additional file 9 Additional file 11). A possible explanation for this result may be that the all genes in the networks were interactional and all the genes in CASN interacted with the seed genes which were all cancer-related genes. Genes that interact often participate in common functional modules like protein complexes, molecular pathways and developmental processes; so, the rest potential genes may have some functions that are similar to the cancer genes.

### The expression level of the optimized genes

**Figure 7:**
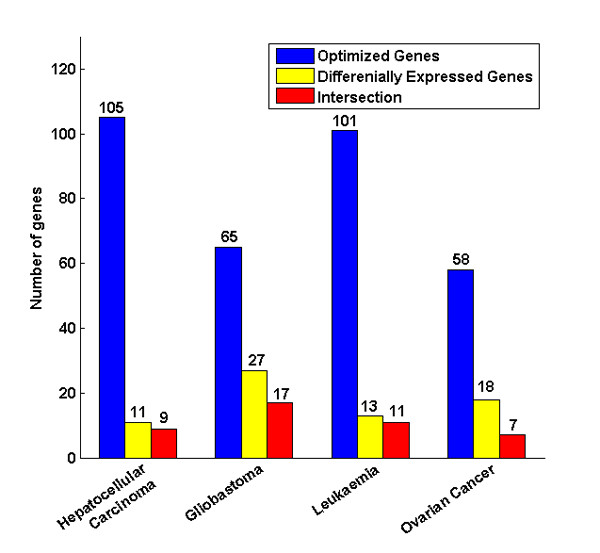
**Comparison of the optimized genes and the differentially expressed genes**. Four types of cancers, hepatocellular carcinoma, glioblastoma, leukemia, and ovarian cancer are on the x axis. The y axis represents the number of genes. The optimized genes are blue and the differentially expressed genes are yellow; the intersection between optimized genes and differentially expressed genes is red.

The expression profiling data for the four types of cancers were obtained from NCBI GEO (See Methods for details). The differentially expressed genes were screened in the expression profiles for the four types of cancers by SAM [35]. The candidate differentially expressed genes that appear in more than 900 re-sampling differentially expressed gene sets were identified as the differentially expressed genes (See Methods for details). This process helped us to identify and remove the numerous insignificant differentially expressed genes. The resultant set contained 52 differentially expressed genes that were ranked by the SAM score *diff*_*score_i _*(Additional file 12). Comparing the differentially expressed genes with the corresponding optimized genes for each of the cancer types (Figure 7), we found that, for the hepatocellular carcinoma expression profiles, 9 of the 11 differentially expressed genes overlapped with our set of 105 optimized genes; for the glioblastoma expression profiles, of the 27 differentially expressed genes, 17 of them overlapped with our set of 65 optimized genes; for the leukemia expression profiles, there were 13 differentially expressed genes, 11 genes were contained in the set of 101 optimized genes; and for the ovarian cancer expression profiles, only 7 of 18 differentially genes were contained in the set of 58 optimized genes.

These findings suggest that some of the optimized genes in our data set might be differentially expressed in the corresponding cancers, indicating that these genes may be regulated by aberrant methylation resulting in their involvement in the cancer-related pathways.

### PubMed co-citations for the optimized genes

To evaluate the relationship between the optimized genes and cancers, we queried the PubMed database for publications that contained the combination of optimized gene, type of cancers and hypermethylation/hypomethylation [36]. The results showed that 43 of 154 optimized genes were associated with cancers and aberrant methylation in PubMed (Additional file 13). Of the 43 cancer and aberrant methylation related genes in PubMed, 10 were reported to have aberrant DNA methylation (Table 1 Additional file 14). In the 10 optimized genes that we found reported in the literature to have changes of methylation level in cancers, 5 were cited as possible diagnostic and prognostic markers for cancers respectively, and 4 were recognized as drug targets (Table 1). The promoter region of *MED1 *(mediator complex subunit 1), one of the optimized aberrant methylated gene associated with cancers, was reported to be frequently methylated in ovarian and colorectal cancer cell lines and this had been to result in the low expression of *MED1 *[37]. *MED1 *was also identified as a drug target. *PRKCDBP*, the gene that encodes the delta binding protein, protein kinase C, was reported to be significantly hypomethylated in breast cancers and the expression of the encoded protein was found to be down-regulated in various cancer cell lines [38]. This gene has also been cited as a diagnostic marker for neuroblastoma.

**Table 1:** Genes validated using PubMed literature co-citations

Symbol	Entrez gene ID	Diagnostic marker	prognostic marker	DrugBank
CREBBP*	1387	▲		target
EP300*	2033	▲	⋆	
HIF1A*	3091		⋆	target
PRMT1*	3276	▲	⋆	target
PML*	5371		⋆	
med1*	5469			target
tp63*	8626	▲	⋆	
PRKCDBP*	112464	▲		
MANEAL*	149175			
Rasef*	158158			

The star (*) in the top right corner of the first column represents this gene is methylated aberrantly in cancers validated by literature. The triangle (▲) in the second column represents this gene can be a diagnostic marker for diseases. And the pentagram (⋆) in the third column represents this gene can be a prognostic marker for diseases supported by literature.

We also manually searched PubMed for an association between all the optimized genes and their use as diagnostic or prognostic markers. We found that 27 of the 154 optimized genes were in the literature as diagnostic markers, and 20 optimized genes were in the literature as prognostic markers for breast cancer, nasopharyngeal carcinoma, prostate cancer, and for other non-cancer diseases that included Rubinstein-Taybi syndrome, type II diabetes and familial dysautonomia (Additional file 14). Subsequently, we searched for the optimized genes in the DrugBank database, and found that 31 genes were annotated as the targets for drug markers (Additional file 14)[39]. Therefore, we conclude that the promoter regions of the optimized genes may be methylated aberrantly, leading to the activation or inhibition of gene expression and contributing to their involvement in the development and progression of cancers.

## Discussion

Here, we prioritized potential cancer-related genes with aberrant methylation based on the constructed weighted human protein-protein interaction network. Network theory has been applied widely to the study of diseases [19]. As a method of describing the interactions between biological molecules, biological network theory reveals the processes and laws involved in, for example, growth, development, aging, and disease. Proteins play important roles in the activity of cells and protein-protein interactions are the main path by which proteins function. Many researchers have used network theory to construct protein-protein interaction networks and combined them with other characteristics, such as molecular pathways and GO annotations, to identify disease-related genes [19]. However, a few studies have attempted to combine network theory with both epigenetic and genetic characteristics [21]. DNA methylation is an important epigenetic modification that influences a variety of physiological activities of the cell, such as X chromosome inactivation, aging, temporal and spatial expression and the development of diseases [40]. Aberrant DNA methylation can affect tumor formation by affecting the chromatin structure and the expression of oncogenes and tumor suppressor genes [41,42]. Therefore, we prioritize the potential cancer-related genes with aberrant methylation by integrating epigenetic and genetic characteristics based on network theory.

In this study, we ensured the reliability of the linkages in the WHPN by deriving the interaction data from five different PPI databases and by selecting only the PPIs which were validated by experiments or which have been reported in the literature. We then built the cancer-associated subnetwork (CASN) from WHPN. The network topological features evaluated using r values related to power law distribution indicate that WHPN is a typical scale-free network. Most biological networks constructed so far are scale-free networks with a degree distribution that follows the power law with degree exponents in the range 2 < r < 3 [23]. Although r_CASN _is below this range, CASN still follows power law distribution and has the characteristics of a scale-free network that has a few Hub nodes with high connectivity and most nodes with low clustering coefficients. Thus, CASN is an approximate scale-free network. This characteristic may be due to the synergetic effect of DNA methylation and the cancer-related seed genes that were used to construct it. This also causes the network structure of CASN to be closer than that of WHPN. The clustering coefficient for CASN (0.1410) is more than the clustering coefficient for WHPN (0.0503). Therefore, in CASN, the genes connected with the seed genes may participate in the same or similar biological processes as the seed genes and may have the same or similar functions as the seed genes. Thus, it can be concluded that the deregulated DNA methylation may affect the development and progression of cancers.

We prioritized 154 potential cancer-related genes with aberrant methylation using the neighborhood-weighting decision rule based on CASN. In the PubMed co-citations analysis for these 154 genes, we found 43 genes that were associated with cancers and aberrant methylation in PubMed. The PubMed co-citations analysis is only a rough assessment which may have a high rate of false positives [36]. Of the 43 genes in the PubMed co-citations, only 10 could be confidently validated to be aberrantly methylated in cancers from the literature analysis. The optimized genes that were not identified in the PubMed co-citations may be cancer-related genes with aberrant methylation that are, as yet, undetected. These genes are prime candidates for validation by further experiments.

## Conclusions

In this study, we constructed a weighted human protein-protein interaction network (WHPN). Based on WHPN, a cancer-association subnetwork (CASN) was obtained using a set of seed genes derived from PubMeth. Comparing the topological features of the two networks, we found that CASN had a much denser network community than WHPN, indicating that the genes in CASN might be aberrantly methylated in cancers and likely participated in the same or similar biological processes as the seed genes. 154 genes were prioritized as potential cancer-related genes with aberrant methylation based on neighborhood-weighting decision rule. The enrichment analysis of GO and KEGG showed that the prioritized genes were generally enriched for biological processes related to apoptosis and programmed cell death and for pathways associated with cancers. Many of the optimized genes showed some degree of differential expression in the SAM analysis, revealing that these genes might be abnormally methylated in the cancer-related biological processes. Abnormal methylation would affect the expression level of these identified genes leading to the development and progression of cancers. In this study, we prioritized the cancer-related genes with aberrant methylation by integrating DNA methylation and protein-protein interaction characteristics based on the network theory. This method will be helpful for the further understanding of the mechanisms of the development and progression in cancers and may help develop new avenues for the prioritization of cancer-related genes with aberrant methylation for diagnosis and therapeutics.

## Methods

### Datasets

#### Protein-protein interaction data

The protein-protein interaction data were obtained from five PPI databases, the Human Protein Reference Database (HPRD) [43], IntAct [44], the Database of Interacting Proteins (DIP) [45], the Molecular INTeraction Database (MINT) [46] and the Biomolecular Interaction Network Database (BIND) [47]. To assure the reliability of the protein-protein interactions, we used only the interaction data that was supported by experimental evidence and not the optimized interactions. Because the different databases use different identifiers, the original identifiers were mapped to the corresponding Entrez Gene IDs using the cross-reference files from Entrez Gene, the HUGO Gene Nomenclature Committee (HGNC) [48] or Biomart [49]. Using this method we obtained 80496 pairs of human protein-protein interaction involving 14611 genes.

#### DNA methylation data

The genome-wide DNA methylation data sources were derived from patients with four types of cancer, glioblastoma (GBM), ovarian cancer, hepatocellular carcinoma and leukemia. The methylation data for glioblastoma and ovarian cancer were from TCGA (The Cancer Genome Atlas) which contained 26 samples for GBM and 394 samples for ovarian cancer. The methylation data for hepatocellular carcinoma and leukemia were obtained from ENCODE in the UCSC Genome Browser which contained two replicates of the HepG2 cell line and two replicates of the K562 cell line. These data were all generated using Infinium assays on the Illumina Infinium HumanMethylation27 BeadChip. In these assays, quantitative measurements of DNA methylation are made for 27578 interrogated CpG dinucleotides covering a genome-wide scale of 14475 genes at single-nucleotide resolution. The probes for the CpG sites were mapped to the corresponding genes using Entrez Gene IDs as the unique identifiers. For genes containing two or more CpG sites, the average methylation value of these CpG sites was used to represent the methylation level of the corresponding gene. Finally, the methylation values of the 27578 CpG sites were mapped to the 14475 genes using the Entrez Gene IDs.

#### Seed gene data

Seed genes were defined as the genes validated by experiments to be aberrantly methylated (hypermethylation or hypomethylation) in cancers. PubMeth, a database of methylation in cancer, contains genes reported to be abnormally methylated in various cancer types. PubMeth is based on automated literature mining which is then manually checked and annotated [50]. We used the keywords, glioblastoma, ovarian cancer, hepatocellular carcinoma and leukemia, in the PubMeth cancer-centric search tool to extract cancer-associated genes with aberrant methylation from the database. After removing the genes for which the number of samples or the methylation frequency was 0, the remaining set of genes was chosen as seed genes. The selected seed gene set included 73 genes for glioblastoma, 58 for hepatocellular carcinoma, 47 for ovarian cancer and 22 for leukemia. These genes were then combined giving a final seed gene set of 127 genes for the four cancer types.

### Construction of WHPN, the weighted human PPI network

We build the weighted network used in this study by combining the human PPI data with the DNA methylation data. Pearson correlations were calculated to assess the association of the methylation for each gene pair. The genes were used as the nodes, the interactions of the gene pairs were the edges and the correlation coefficient was used as the linkage weight. Linkages for which the methylation correlations were beneath the threshold were removed.

Next, the weight thresholds of the network were determined. The DNA methylation data was perturbed for 1000 times; thereafter, the Pearson correlation coefficient of the random DNA methylation was computed for the gene pairs in each perturbed methylation dataset. The methylation correlation of the original gene pairs was compared with the methylation correlations of the perturbed gene pairs. All the values were ranked and compared with the true methylation correlation. If the true value was either in the top five percent or in the bottom five percent of the ranked correlations for the perturbed gene pairs (P value < 0.1), then the methylation correlation for that pair was considered to be significant and this correlation was used as the linkage weight of the connected gene pair. All gene pairs for which the methylation correlations were not within the top or bottom five percent were removed from the PPI network.

### Construction of CASN, the cancer-associated subnetwork

The seed gene set obtained using the data from PubMeth was mapped into the weighted human PPI network. Thus, the cancer-associated subnetwork (CASN) is composed of the seed genes and the genes that are connected with the seed genes in the weighted PPI network.

### Network randomization

Random subnetworks were generated by randomly sampling the same number of nodes as in CASN from WHPN; the connections in the sampling nodes were kept in the random subnetworks. This process was repeated 1000 times. Topology-matched random subnetworks were generated by the method described by Li et al. in an earlier study [51]. Similarly, all the genes in WHPN were divided into three subsets of equal size based on degrees and clustering coefficients. Next, the WHPN genes were assigned to nine sets by combining the three degree sets with the three clustering coefficient sets. To create the random subnetwork, for every node in CASN, we randomly obtained one node from the same topological set in WHPN; in this way, 1000 topology-matched random subnetworks were generated. Finally, to determine the significance of our results, a third randomization method was used to generate 1000 random subnetworks by keeping the number of nodes and connections of CASN.

### Network visualization

The networks were drawn using Cytoscape http://www.cytoscape.org/, an open source software platform for visualizing complex biological networks [52].

### The calculation of topological features in the two networks

Network theory provides a quantifiable description of networks for the biological systems. And degree, clustering coefficient and average path length are the three characteristic topological features used to measure complex networks. In this study, we examined these topological features that can be used to measure and compare the different complex networks [23].

#### Degree

The degree (connectivity) of a node i, *k_i_*, is defined as the number of notes which the node i connects. And the average degree of the network is the average value of the degrees of all the notes, 2L/N, where L represents links and N represents notes.

#### Degree distribution

This measurement indicates that the variety trend of probability for the note whose degree is k with the change of the note's degree k, and it also means that the ratio of the number of notes with the degree of k accounting for all the notes in the network.

#### Scale-free networks

Most biological networks are scale-free whose degree distribution follows a power law, at least asymptotically. That is,

(1) $$P\left(k\right)\sim k^{-r}$$

r is the index for the distribution. According to

(2) $$p\left(k\right)=ak^{-r}$$

The r values for the networks are calculated, looking for whether they asymptotically follow power law distribution.

#### Clustering coefficients

If the degree of a note i is *k_i _*and the edge number of the linked neighbor notes is *E_i_*, the clustering coefficient of the note i is

(3) $$C_{i}=2E_{i}∕\left(k_{i}\left(k_{i}-1\right)\right)$$

The clustering coefficient of the network is defined as the average value for clustering coefficient of all the notes, which reflects the overall cluster trend of interacted notes.

#### Average path length

In the network research, the general definition of the distance between two nodes is the shortest path connecting the two notes, and the network diameter is the maximum distance between any two notes, that is

(4) $$D=\underset{i,j}{\max}d_{ij}$$

The average path of the network < L > is the average value of the distances for all notes,

(5) $$L=\frac{1}{\frac{1}{2}N\left(N-1\right)}\underset{i\geq j}{\sum }d_{ij}$$

### Subnetwork-based cancer gene with aberrant methylation prioritization

#### Neighborhood-weighting decision rule

Candidate genes are defined as the genes that connect to seed genes [20]. Given the seed gene set of cancers, the candidate gene i associated with the seed genes in the subnetwork are quantitatively measured using score *S_i_*:

(6) $$S_{i}=\sum |\omega_{ij}|$$

*ω_ij _*represents the linkage weight that between the candidate gene i and the seed gene j, viz. the Pearson correlation of the DNA methylation between the two genes. If the candidate gene i is not associated with the seed gene j, *ω_ij _*= 0. The scores of the candidate genes are calculated by the neighborhood-weighting decision rule.

And next, the candidate genes are prioritized according to the score *S_i_*. Through the perturbation of DNA methyaltion data, the permuted weights for linkages are obtained. And then the permuted scores are also calculated by the Neighborhood-weighting decision rule. This procedure is repeated 1000 times and then the scores and the permuted scores are compared. The genes whose score is grater than all the corresponding permuted score are selected. It is believe that these genes have potential to be methylated aberrantly in cancers.

### Enrichment analysis of GO and KEGG pathway

The gene annotation enrichment analysis for the optimized genes was taken by DAVID [53], where the tools can provide functional interpretation of large gene lists derived from genomic studies.

### Comparisons of differentially expressed genes

Using the gene expression profiles from NCBI GEO (GSE4290, GSE14811, GSE5788 and GSE18520), the profiles of cancer samples and control samples were compared to find differentially expressed genes by SAM method [35]. The genomes of tumor cells are usually more unstable than the genomes of other cells. In an earlier study, Li et al. [54] speculated that there may be many genes with variable expression between individual tumor cells making the differentially expressed genes that are really tumor-specific hard to find in the highly varied profiles. To overcome this problem, we firstly identified candidate differentially expressed genes using the original NCBI GEO dataset. The cancer and control samples in the datasets were re-sampled 1000 times taking care to maintain the same ratio of control to cancer samples as in the original dataset. Then, for each random dataset, the apparently differentially expressed genes were identified. For each candidate differentially expressed gene i, *n_i_*, the number of times this gene appeared in the 1000 random differentially expressed gene sets was calculated. If *n_i _*was > 900, then gene i was identified as a differentially expressed gene and the *diff*_*score_i _*for this gene was defined as,

(7) diff_scorei=∑j=11000dijni

where, *d_ij _*is the SAM score for gene i in the random dataset j. The differentially expressed genes were ranked according to their *diff*_*score_i_*. SAM was performed using the R package samr.

### Text-mining of PubMed for the optimized genes comparison to previously publications

In the absence of a gold standard to assess the relationship between the cancers and the methylation level of the optimized genes, we used the public database PubMed to produce the reference lists. PubMed contains hundreds of thousands of citations to the biomedical literature. We systematically searched PubMed using query terms that included the optimized gene name, the cancer type and hypermethylation/hypomethylation, for the co-occurrence of these terms either in an abstract or in the title of previous publications [36].

## Abbreviations

WHPN: Weighted Human Protein-protein interaction Network; CASN: Cancer-associated Subnetwork; GO: Gene Ontology; KEGG: Kyoto Encyclopedia of Genes and Genomes; PPI: Protein-protein Interaction; HPRD: Human Protein Reference Database; DIP: Database of Interacting Proteins; MINT: INTeraction Database; BIND: Biomolecular Interaction Network Database; HGNC: the HUGO Gene Nomenclature Committee; TCGA: the Cancer Genome Atlas; ENCODE: ENCyclopedia of DNA Elements; GEO: Gene Expression Omnibus; DAVID: the Database for Annotation, Visualization and Integrated Discovery; SAM: Significance Analysis of Microarrays.

## Authors' contributions

YZ and HQ conceived and designed the experiments. HL, JL and BL acquired the experiment data. HL, JS and HL performed the study. HL, JS, HL and JL carried out the data analysis. HL wrote this manuscript. All authors have read and approved the final manuscript.

## Supplementary Material

Additional file 1 **Category of seed genes**. The first column is the Entrez Gene ID for the seed genes. In columns 2 to 5, 1 represents a seed gene that is related with this type of cancers and 0 represents a seed gene that is not related with this type of cancers. The last column shows the types of the seed genes. The seed genes were classified into 4 types; genes related with one type of cancers are marked with 1, genes related with two types of cancers are marked with 2, genes related with three types of cancers are marked with 3, and the genes related with all the four types of cancers are marked with 4. Click here for file

Additional file 2 **Comparison of CASN and random subnetworks**. Comparison between degree and clustering coefficient of CASN and the three kinds of random subnetworks. Click here for file

Additional file 3 **Scores of the optimized genes**. The first column is the Entrez Gene ID for the optimized gene. The second and the third columns are the true and random scores respectively, for the optimized genes by the neighborhood-weighting decision rule. Click here for file

Additional file 4 **Category of optimized genes**. The first column is the Entrez Gene ID for the optimized gene. From columns 2 to 5, 1 represents an optimized gene that interacts with the seed genes related with the type of cancers and 0 represents an optimized gene that does not interact with the seed genes related with the type of cancers. The last column shows the types of the optimized genes. The optimized genes were classified into 4 types, the genes related with one type of cancer are marked with 1, the genes related with two types of cancers are marked with 2, the genes related with three types of cancers are marked with 3, and the genes related with all the four types of cancers are marked with 4. Click here for file

Additional file 5 **Degree of the genes in WHPN and CASN**. The genes are on the x axes and the degree of the genes is on the y axes. (A) The degrees of the CASN genes (red dots) and non-CASN genes (blue dots) in WHPN; (B) The degrees of the seed genes (red dots), optimized genes (blue dots) and rest potential genes (yellow dots) in CASN. Click here for file

Additional file 6 **Comparison of CASN and non-CASN genes**. Comparisons are based on mean, median, minimum, maximum and the percentiles 25, 50 and 75. Click here for file

Additional file 7 **Comparison of seed genes, optimized genes and rest potential genes**. Comparisons are based on mean, median, minimum, maximum and the percentiles 25, 50 and 75. Click here for file

Additional file 8 **GO enrichment analysis for WHPN genes**. The GO enrichment analysis of CASN genes and non-CASN genes in WHPN are shown in Additional file 8. A P value of < 0.05 was taken to be significant. Click here for file

Additional file 9 **GO enrichment analysis for CASN genes**. The GO enrichment analysis of seed genes, optimized genes and rest potential genes are shown in Additional file 9. A P value of < 0.05 was taken to be significant. Click here for file

Additional file 10 **KEGG enrichment analysis for WHPN genes**. The KEGG enrichment analysis of CASN genes and non-CASN genes in WHPN are shown in Additional file 10. A P value of < 0.05 was taken to be significant. Click here for file

Additional file 11 **KEGG enrichment analysis for CASN genes**. The KEGG enrichment analysis of seed genes, optimized genes and rest potential genes are shown in Additional file 11. A P value of < 0.05 was taken to be significant. Click here for file

Additional file 12 **SAM score for the differentially expressed genes**. Of the 154 optimized genes, 52 differentially expressed genes and they are ranked by their *diff*_*score_i_*. Click here for file

Additional file 13 **PubMed co-citations of the optimized genes**. The first column is the Entrez Gene ID for the optimized genes. Of the 154 optimized genes, 43 genes that were found from a preliminary analysis to be associated with cancers and aberrant methylation in PubMed. The star (*) in the first column marks a gene that was subsequently validated to be methylated aberrantly in cancers by text mining the literature in PubMed. Click here for file

Additional file 14 **Diagnostic, prognostic and drug marker validation of optimized genes**. After searching PubMed manually, 27 genes were identified as diagnostic markers and 20 genes were identified as prognostic markers for cancers and other complex diseases. Mapped into DrugBank target list, 31 genes can be target as drug response markers. Click here for file
