# Supplementary figures and images for: Prioritizing cancer-related genes with aberrant methylation based on a weighted protein-protein interaction network
Source: BMC Syst Biol. 2011 Oct 11;5:158. doi: 10.1186/1752-0509-5-158 (PMC3224234; doi:10.1186/1752-0509-5-158)

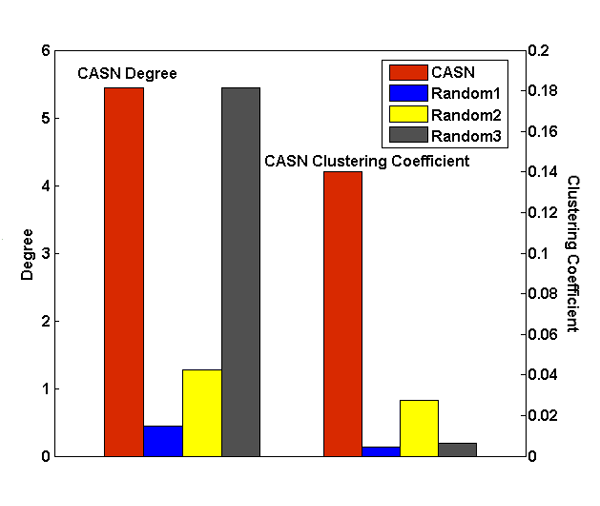

Supplement: Additional file 2 — Comparison of CASN and random subnetworks. Comparison between degree and clustering coefficient of CASN and the three kinds of random subnetworks. [file 1752-0509-5-158-S2.PNG]

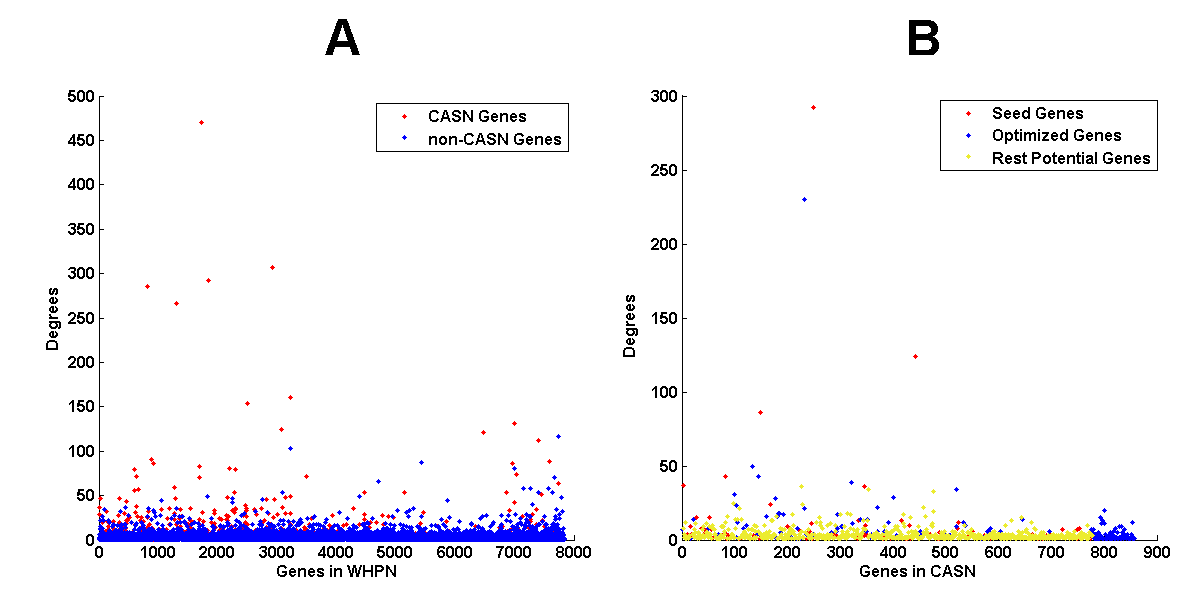

Supplement: Additional file 5 — Degree of the genes in WHPN and CASN. The genes are on the x axes and the degree of the genes is on the y axes. (A) The degrees of the CASN genes (red dots) and non-CASN genes (blue dots) in WHPN; (B) The degrees of the seed genes (red dots), optimized genes (blue dots) and rest potential genes (yellow dots) in CASN. [file 1752-0509-5-158-S5.PNG]
