# Supplementary material for: Prioritizing cancer-related genes with aberrant methylation based on a weighted protein-protein interaction network
Source: BMC Syst Biol. 2011 Oct 11;5:158. doi: 10.1186/1752-0509-5-158 (PMC3224234; doi:10.1186/1752-0509-5-158)
Supplement: Additional file 14 — Diagnostic, prognostic and drug marker validation of optimized genes. After searching PubMed manually, 27 genes were identified as diagnostic markers and 20 genes were identified as prognostic markers for cancers and other complex diseases. Mapped into DrugBank target list, 31 genes can be target as drug response markers. [file 1752-0509-5-158-S14.DOC]

| Symbol | GeneID | Disease | Diagnostic  Marker | Prognostic  Marker | drug response marker |
| --- | --- | --- | --- | --- | --- |
| *CCND3* | 896 | bladder carcinoma, Pancreatic adenocarcinoma,  ovarian adenocarcinomas, hematolymphoid neoplasms,  rimary Ta/T1 bladder cancer | [1-2] | [3-5] |  |
| *CD69* | 969 | chronic lymphocytic leukemia，  melanoma,  head and neck squamous cell carcinoma | [6] | [7-9] |  |
| *CREBBP* | 1387 | Rubinstein-Taybi syndrome,  cancer,  melanoma,  primary cutaneous | [10-11] |  | Target |
| *e2f4* | 1874 | salivary gland tumors， breast carcinogenesis | [12] | [12-13] |  |
| *EP300* | 2033 | colorectal adenocarcinoma,  hepatocellular carcinoma | [11, 14] | [15] |  |
| *PRMT1* | 3276 | Hirschsprung disease，breast cancer | [16] | [17] | Target |
| *mdm2* | 4193 | low-grade osteosarcomas, nasopharyngeal carcinoma | [18-20] |  | Target |
| *RNASEL* | 6041 | carcinoma of the uterine cervix,  HNSCC,  breast cancer，  Prostate Cancer | [21] | [22] |  |
| *A2M* | 2 |  |  |  | Target |
| *Skp2* | 6502 | human melanoma, Non-small cell lung cancer，  nasopharyngeal carcinoma，  diffuse large B-cell lymphoma | [23-24] | [25-26] |  |
| *Tsg101* | 7251 | breast cancer | [27] |  |  |
| *IKBKAP* | 8518 | [familial dysautonomia.](http://www.ncbi.nlm.nih.gov/pubmed/18091349) | [28-29] |  |  |
| *tp63* | 8626 | Prostate cancer，  node negative breast cancer | [30] | [31] |  |
| *PRKCDBP* | 112464 | neuroblastoma. | [32] |  |  |
| *Rassf3* | 283349 | cancer | [33] |  |  |
| *Apex1* | 328 | prostate cancer,  osteosarcoma | [34] | [35] | Target |
| *DDX5* | 1655 | multiple myeloma tumorigenesis | [36] |  |  |
| *Hnf4a* | 3172 | type 2 diabetes，colorectal carcinoma | [37] | [38] | Target |
| *PDE4D* | 5144 | prostate cancer | [39] |  | Target |
| *Rnf4* | 6047 | colorectal cancers | [40] |  |  |
| *ROS1* | 6098 | cholangiocarcinoma | [41] |  |  |
| *TRIM24* | 8805 | breast cancer | [42] | [43] |  |
| *Sirpa* | 140885 | breast cancer |  | [44] |  |
| *MIR17HG* | 407975 | alveolar rhabdomyosarcoma |  | [45] |  |
| *csf1r* | 1436 | hepatocellular carcinoma |  | [46] | Target |
| *Hdac1* | 3065 | AML |  | [47] | Target |
| *sp1* | 6667 | glioma |  | [48] |  |
| *tubB* | 203068 | head and neck squamous cell carcinoma |  | [49] | Target |
| *ST5* | 6764 | diffuse large B-cell lymphoma |  | [50] |  |
| *TP53RK* | 112858 | MCF7 cancer cell line | [51] |  |  |
| *NCOA7* | 135112 | breast cancer | [52] |  |  |
| *TMPRSS6* | 164656 | breast cancer, iron-refractory iron deficiency anemia | [53-54] |  |  |
| *Fam129c* | 199786 | colorectal cancer | [55] |  |  |
| *STRA13* | 201254 | renal carcinomas | [56] |  |  |
| *SMARCA4* | 6597 | atypical teratoid/rhabdoid tumor | [57] |  |  |
| *PML* | 5371 | esophageal squamous cell carcinomas，ampullary cancer，gallbladder carcinomas |  | [58-60] |  |
| *anxa3* | 306 |  |  |  | Target |
| *HIF1A* | 3091 | esophageal squamous cell carcinoma. |  | [61] | Target |
| *cast* | 831 |  |  |  | Target |
| *CSNK2A1P* | 1457 |  |  |  | Target |
| *HSP90AA2* | 3320 |  |  |  | Target |
| *Mttp* | 259 |  |  |  | Target |
| *PLD1* | 5337 |  |  |  | Target |
| *plg* | 5340 |  |  |  | Target |
| *RXRA* | 6256 |  |  |  | Target |
| *Rxrb* | 6257 |  |  |  | Target |
| *TUBA4A* | 7277 |  |  |  | Target |
| *Grin3a* | 116443 |  |  |  | Target |
| *SLC44A3* | 126969 |  |  |  | Target |
| *KAT2B* | 8850 |  |  |  | Target |
| *Syt2* | 127833 |  |  |  | Target |
| *SLC36A1* | 206358 |  |  |  | Target |
| *med1* | 5469 |  |  |  | Target |
| *Mapk8* | 5599 |  |  |  | Target |
| *NCOA1* | 8648 |  |  |  | Target |
| *DCN* | 1634 |  |  |  | Target |
| *Mapk9* | 6274 |  |  |  | Target |

**References**

1. Lopez-Beltran A, Ordonez JL, Otero AP, Blanca A, Sevillano V, Sanchez-Carbayo M, Munoz E, Cheng L, Montironi R, de Alava E: **Cyclin D3 gene amplification in bladder carcinoma in situ.** *Virchows Arch* 2010, **457:**555-561.

2. Birnbaum DJ, Adelaide J, Mamessier E, Finetti P, Lagarde A, Monges G, Viret F, Goncalves A, Turrini O, Delpero JR, et al: **Genome profiling of pancreatic adenocarcinoma.** *Genes Chromosomes Cancer* 2011, **50:**456-465.

3. Metcalf RA, Zhao S, Anderson MW, Lu ZS, Galperin I, Marinelli RJ, Cherry AM, Lossos IS, Natkunam Y: **Characterization of D-cyclin proteins in hematolymphoid neoplasms: lack of specificity of cyclin-D2 and D3 expression in lymphoma subtypes.** *Mod Pathol* 2010, **23:**420-433.

4. Levidou G, Korkolopoulou P, Thymara I, Vassilopoulos I, Saetta AA, Gakiopoulou H, Konstantinidou A, Kairi-Vassilatou E, Pavlakis K, Patsouris E: **Expression and prognostic significance of cyclin D3 in ovarian adenocarcinomas.** *Int J Gynecol Pathol* 2007, **26:**410-417.

5. Lopez-Beltran A, Requena MJ, Luque RJ, Alvarez-Kindelan J, Quintero A, Blanca AM, Rodriguez ME, Siendones E, Montironi R: **Cyclin D3 expression in primary Ta/T1 bladder cancer.** *J Pathol* 2006, **209:**106-113.

6. Petersen CC, Nederby L, Roug AS, Skovbo A, Peterslund NA, Hokland P, Nielsen B, Hokland M: **Increased expression of CD69 on T cells as an early immune marker for human cytomegalovirus reactivation in chronic lymphocytic leukemia patients.** *Viral Immunol* 2011, **24:**165-169.

7. Hernberg M, Mattila PS, Rissanen M, Hansson J, Aamdal S, Bastholt L, von der Maase H, Schmidt H, Stierner U, Tarkkanen J: **The prognostic role of blood lymphocyte subset distribution in patients with resected high-risk primary or regionally metastatic melanoma.** *J Immunother* 2007, **30:**773-779.

8. Hillen F, Baeten CI, van de Winkel A, Creytens D, van der Schaft DW, Winnepenninckx V, Griffioen AW: **Leukocyte infiltration and tumor cell plasticity are parameters of aggressiveness in primary cutaneous melanoma.** *Cancer Immunol Immunother* 2008, **57:**97-106.

9. Badoual C, Hans S, Rodriguez J, Peyrard S, Klein C, Agueznay Nel H, Mosseri V, Laccourreye O, Bruneval P, Fridman WH, et al: **Prognostic value of tumor-infiltrating CD4+ T-cell subpopulations in head and neck cancers.** *Clin Cancer Res* 2006, **12:**465-472.

10. Sharma N, Mali AM, Bapat SA: **Spectrum of CREBBP mutations in Indian patients with Rubinstein-Taybi syndrome.** *J Biosci* 2010, **35:**187-202.

11. Tillinghast GW, Partee J, Albert P, Kelley JM, Burtow KH, Kelly K: **Analysis of genetic stability at the EP300 and CREBBP loci in a panel of cancer cell lines.** *Genes Chromosomes Cancer* 2003, **37:**121-131.

12. Russo G, Zamparelli A, Howard CM, Minimo C, Bellan C, Carillo G, Califano L, Leoncini L, Giordano A, Claudio PP: **Expression of cell cycle-regulated proteins pRB2/p130, p107, E2F4, p27, and pCNA in salivary gland tumors: prognostic and diagnostic implications.** *Clin Cancer Res* 2005, **11:**3265-3273.

13. Rakha EA, Pinder SE, Paish EC, Robertson JF, Ellis IO: **Expression of E2F-4 in invasive breast carcinomas is associated with poor prognosis.** *J Pathol* 2004, **203:**754-761.

14. Ishihama K, Yamakawa M, Semba S, Takeda H, Kawata S, Kimura S, Kimura W: **Expression of HDAC1 and CBP/p300 in human colorectal carcinomas.** *J Clin Pathol* 2007, **60:**1205-1210.

15. Li M, Luo RZ, Chen JW, Cao Y, Lu JB, He JH, Wu QL, Cai MY: **High expression of transcriptional coactivator p300 correlates with aggressive features and poor prognosis of hepatocellular carcinoma.** *J Transl Med* 2011, **9:**5.

16. Wu TT, Tsai TW, Shen YT, Hsu JD, Yang LC, Li C: **Analyses of PRMT1 proteins in human colon tissues from Hirschsprung disease patients.** *Neurogastroenterol Motil* 2010, **22:**984-990, e254.

17. Mathioudaki K, Scorilas A, Ardavanis A, Lymberi P, Tsiambas E, Devetzi M, Apostolaki A, Talieri M: **Clinical evaluation of PRMT1 gene expression in breast cancer.** *Tumour Biol* 2011, **32:**575-582.

18. Wan Y, Wu W, Yin Z, Guan P, Zhou B: **MDM2 SNP309, gene-gene interaction, and tumor susceptibility: an updated meta-analysis.** *BMC Cancer* 2011, **11:**208.

19. Dujardin F, Binh MB, Bouvier C, Gomez-Brouchet A, Larousserie F, Muret A, Louis-Brennetot C, Aurias A, Coindre JM, Guillou L, et al: **MDM2 and CDK4 immunohistochemistry is a valuable tool in the differential diagnosis of low-grade osteosarcomas and other primary fibro-osseous lesions of the bone.** *Mod Pathol* 2011, **24:**624-637.

20. Sousa H, Pando M, Breda E, Catarino R, Medeiros R: **Role of the MDM2 SNP309 polymorphism in the initiation and early age of onset of nasopharyngeal carcinoma.** *Mol Carcinog* 2011, **50:**73-79.

21. Madsen BE, Ramos EM, Boulard M, Duda K, Overgaard J, Nordsmark M, Wiuf C, Hansen LL: **Germline mutation in RNASEL predicts increased risk of head and neck, uterine cervix and breast cancer.** *PLoS One* 2008, **3:**e2492.

22. Lin DW, Fitzgerald LM, Fu R, Kwon EM, Zheng SL, Kolb S, Wiklund F, Stattin P, Isaacs WB, Xu J, et al: **Genetic Variants in the LEPR, CRY1, RNASEL, IL4, and ARVCF Genes Are Prognostic Markers of Prostate Cancer-Specific Mortality.** *Cancer Epidemiol Biomarkers Prev* 2011.

23. Chen G, Cheng Y, Zhang Z, Martinka M, Li G: **Cytoplasmic Skp2 expression is increased in human melanoma and correlated with patient survival.** *PLoS One* 2011, **6:**e17578.

24. Jiang F, Todd NW, Li R, Zhang H, Fang H, Stass SA: **A panel of sputum-based genomic marker for early detection of lung cancer.** *Cancer Prev Res (Phila)* 2010, **3:**1571-1578.

25. Xu HM, Liang Y, Chen Q, Wu QN, Guo YM, Shen GP, Zhang RH, He ZW, Zeng YX, Xie FY, Kang TB: **Correlation of Skp2 overexpression to prognosis of patients with nasopharyngeal carcinoma from South China.** *Chin J Cancer* 2011, **30:**204-212.

26. Seki R, Ohshima K, Fujisaki T, Uike N, Kawano F, Gondo H, Makino S, Eto T, Moriuchi Y, Taguchi F, et al: **Prognostic significance of S-phase kinase-associated protein 2 and p27kip1 in patients with diffuse large B-cell lymphoma: effects of rituximab.** *Ann Oncol* 2010, **21:**833-841.

27. Lee MP, Feinberg AP: **Aberrant splicing but not mutations of TSG101 in human breast cancer.** *Cancer Res* 1997, **57:**3131-3134.

28. Gold-von Simson G, Leyne M, Mull J, Rolnitzky LM, Goldberg JD, Berlin D, Axelrod FB, Slaugenhaupt SA: **IKBKAP mRNA in peripheral blood leukocytes: a molecular marker of gene expression and splicing in familial dysautonomia.** *Pediatr Res* 2008, **63:**186-190.

29. Slaugenhaupt SA, Blumenfeld A, Gill SP, Leyne M, Mull J, Cuajungco MP, Liebert CB, Chadwick B, Idelson M, Reznik L, et al: **Tissue-specific expression of a splicing mutation in the IKBKAP gene causes familial dysautonomia.** *Am J Hum Genet* 2001, **68:**598-605.

30. Yaskiv O, Zhang X, Simmerman K, Daly T, He H, Falzarano S, Chen L, Magi-Galluzzi C, Zhou M: **The utility of ERG/P63 double immunohistochemical staining in the diagnosis of limited cancer in prostate needle biopsies.** *Am J Surg Pathol* 2011, **35:**1062-1068.

31. Gudlaugsson E, Skaland I, Undersrud E, Janssen EA, Soiland H, Baak JP: **D2-40/p63 defined lymph vessel invasion has additional prognostic value in highly proliferating operable node negative breast cancer patients.** *Mod Pathol* 2011, **24:**502-511.

32. Caren H, Djos A, Nethander M, Sjoberg RM, Kogner P, Enstrom C, Nilsson S, Martinsson T: **Identification of epigenetically regulated genes that predict patient outcome in neuroblastoma.** *BMC Cancer* 2011, **11:**66.

33. Richter AM, Pfeifer GP, Dammann RH: **The RASSF proteins in cancer; from epigenetic silencing to functional characterization.** *Biochim Biophys Acta* 2009, **1796:**114-128.

34. Kuasne H, Rodrigues IS, Losi-Guembarovski R, Reis MB, Fuganti PE, Gregorio EP, Libos Junior F, Matsuda HM, Rodrigues MA, Kishima MO, Colus IM: **Base excision repair genes XRCC1 and APEX1 and the risk for prostate cancer.** *Mol Biol Rep* 2011, **38:**1585-1591.

35. Yang J, Yang D, Cogdell D, Du X, Li H, Pang Y, Sun Y, Hu L, Sun B, Trent J, et al: **APEX1 gene amplification and its protein overexpression in osteosarcoma: correlation with recurrence, metastasis, and survival.** *Technol Cancer Res Treat* 2010, **9:**161-169.

36. Felix RS, Colleoni GW, Caballero OL, Yamamoto M, Almeida MS, Andrade VC, Chauffaille Mde L, Silva WA, Jr., Begnami MD, Soares FA, et al: **SAGE analysis highlights the importance of p53csv, ddx5, mapkapk2 and ranbp2 to multiple myeloma tumorigenesis.** *Cancer Lett* 2009, **278:**41-48.

37. Silander K, Mohlke KL, Scott LJ, Peck EC, Hollstein P, Skol AD, Jackson AU, Deloukas P, Hunt S, Stavrides G, et al: **Genetic variation near the hepatocyte nuclear factor-4 alpha gene predicts susceptibility to type 2 diabetes.** *Diabetes* 2004, **53:**1141-1149.

38. Oshima T, Kawasaki T, Ohashi R, Hasegawa G, Jiang S, Umezu H, Aoyagi Y, Iwanari H, Tanaka T, Hamakubo T, et al: **Downregulated P1 promoter-driven hepatocyte nuclear factor-4alpha expression in human colorectal carcinoma is a new prognostic factor against liver metastasis.** *Pathol Int* 2007, **57:**82-90.

39. Rahrmann EP, Collier LS, Knutson TP, Doyal ME, Kuslak SL, Green LE, Malinowski RL, Roethe L, Akagi K, Waknitz M, et al: **Identification of PDE4D as a proliferation promoting factor in prostate cancer using a Sleeping Beauty transposon-based somatic mutagenesis screen.** *Cancer Res* 2009, **69:**4388-4397.

40. Quyun C, Ye Z, Lin SC, Lin B: **Recent patents and advances in genomic biomarker discovery for colorectal cancers.** *Recent Pat DNA Gene Seq* 2010, **4:**86-93.

41. Gu TL, Deng X, Huang F, Tucker M, Crosby K, Rimkunas V, Wang Y, Deng G, Zhu L, Tan Z, et al: **Survey of tyrosine kinase signaling reveals ROS kinase fusions in human cholangiocarcinoma.** *PLoS One* 2011, **6:**e15640.

42. Allton K, Jain AK, Herz HM, Tsai WW, Jung SY, Qin J, Bergmann A, Johnson RL, Barton MC: **Trim24 targets endogenous p53 for degradation.** *Proc Natl Acad Sci U S A* 2009, **106:**11612-11616.

43. Chambon M, Orsetti B, Berthe ML, Bascoul-Mollevi C, Rodriguez C, Duong V, Gleizes M, Thenot S, Bibeau F, Theillet C, Cavailles V: **Prognostic significance of TRIM24/TIF-1alpha gene expression in breast cancer.** *Am J Pathol* 2011, **178:**1461-1469.

44. Nagahara M, Mimori K, Kataoka A, Ishii H, Tanaka F, Nakagawa T, Sato T, Ono S, Sugihara K, Mori M: **Correlated expression of CD47 and SIRPA in bone marrow and in peripheral blood predicts recurrence in breast cancer patients.** *Clin Cancer Res* 2010, **16:**4625-4635.

45. Reichek JL, Duan F, Smith LM, Gustafson DM, O'Connor RS, Zhang C, Dunlevy MJ, Gastier-Foster JM, Barr FG: **Genomic and clinical analysis of amplification of the 13q31 chromosomal region in alveolar rhabdomyosarcoma: a report from the Children's Oncology Group.** *Clin Cancer Res* 2011, **17:**1463-1473.

46. Jia JB, Wang WQ, Sun HC, Zhu XD, Liu L, Zhuang PY, Zhang JB, Zhang W, Xu HX, Kong LQ, et al: **High expression of macrophage colony-stimulating factor-1 receptor in peritumoral liver tissue is associated with poor outcome in hepatocellular carcinoma after curative resection.** *Oncologist* 2010, **15:**732-743.

47. Tickenbrock L, Klein HU, Trento C, Hascher A, Gollner S, Baumer N, Kuss R, Agrawal S, Bug G, Serve H, et al: **Increased HDAC1 deposition at hematopoietic promoters in AML and its association with patient survival.** *Leuk Res* 2011, **35:**620-625.

48. Guan H, Cai J, Zhang N, Wu J, Yuan J, Li J, Li M: **Sp1 is upregulated in human glioma, promotes MMP-2-mediated cell invasion and predicts poor clinical outcome.** *Int J Cancer* 2011.

49. Koh Y, Kim TM, Jeon YK, Kwon TK, Hah JH, Lee SH, Kim DW, Wu HG, Rhee CS, Sung MW, et al: **Class III beta-tubulin, but not ERCC1, is a strong predictive and prognostic marker in locally advanced head and neck squamous cell carcinoma.** *Ann Oncol* 2009, **20:**1414-1419.

50. Mahadevan D, Spier C, Della Croce K, Miller S, George B, Riley C, Warner S, Grogan TM, Miller TP: **Transcript profiling in peripheral T-cell lymphoma, not otherwise specified, and diffuse large B-cell lymphoma identifies distinct tumor profile signatures.** *Mol Cancer Ther* 2005, **4:**1867-1879.

51. Sarvaiya HA, Yoon JH, Lazar IM: **Proteome profile of the MCF7 cancer cell line: a mass spectrometric evaluation.** *Rapid Commun Mass Spectrom* 2006, **20:**3039-3055.

52. Higginbotham KS, Breyer JP, Bradley KM, Schuyler PA, Plummer WD, Jr., Freudenthal ME, Trentham-Dietz A, Newcomb PA, Sanders ME, Page DL, et al: **A multistage association study identifies a breast cancer genetic locus at NCOA7.** *Cancer Res* 2011, **71:**3881-3888.

53. Hartikainen JM, Tuhkanen H, Kataja V, Eskelinen M, Uusitupa M, Kosma VM, Mannermaa A: **Refinement of the 22q12-q13 breast cancer--associated region: evidence of TMPRSS6 as a candidate gene in an eastern Finnish population.** *Clin Cancer Res* 2006, **12:**1454-1462.

54. Wu HM, Li L, Yuan XW, Zhou YQ, Xiao QZ, Liu WY, Zhou WJ, Xu XM: **Rapid, accurate detection of TMPRSS6 gene causative mutations with a high-resolution melting assay.** *Blood Cells Mol Dis* 2011.

55. Han M, Liew CT, Zhang HW, Chao S, Zheng R, Yip KT, Song ZY, Li HM, Geng XP, Zhu LX, et al: **Novel blood-based, five-gene biomarker set for the detection of colorectal cancer.** *Clin Cancer Res* 2008, **14:**455-460.

56. Ivanova A, Liao SY, Lerman MI, Ivanov S, Stanbridge EJ: **STRA13 expression and subcellular localisation in normal and tumour tissues: implications for use as a diagnostic and differentiation marker.** *J Med Genet* 2005, **42:**565-576.

57. Hasselblatt M, Gesk S, Oyen F, Rossi S, Viscardi E, Giangaspero F, Giannini C, Judkins AR, Fruhwald MC, Obser T, et al: **Nonsense mutation and inactivation of SMARCA4 (BRG1) in an atypical teratoid/rhabdoid tumor showing retained SMARCB1 (INI1) expression.** *Am J Surg Pathol* 2011, **35:**933-935.

58. Yen CC, Tsao YP, Chen PC, Wu YC, Liu JH, Pan CC, Liu CY, Tzeng CH, Chen PM, Chen YJ, et al: **PML protein as a prognostic molecular marker for patients with esophageal squamous cell carcinomas receiving primary surgery.** *J Surg Oncol* 2011, **103:**761-767.

59. Vincenzi B, Santini D, Perrone G, Russo A, Adamo V, Rizzo S, Castri F, Antinori A, Alloni R, Crucitti PF, et al: **Promyelocytic leukemia (PML) gene expression is a prognostic factor in ampullary cancer patients.** *Ann Oncol* 2009, **20:**78-83.

60. Chang HJ, Yoo BC, Kim SW, Lee BL, Kim WH: **Significance of PML and p53 protein as molecular prognostic markers of gallbladder carcinomas.** *Pathol Oncol Res* 2007, **13:**326-335.

61. Munipalle PC, Viswanath YK, Davis PA, Scoones D: **Prognostic value of hypoxia inducible factor 1alpha in esophageal squamous cell carcinoma.** *Dis Esophagus* 2011, **24:**177-181.
